# A Novel Infrared Spectroscopy Method for Analysis of Stone Dust for Establishing Final Composition of Urolithiasis

**DOI:** 10.1016/j.euros.2022.11.007

**Published:** 2022-12-15

**Authors:** Marius Snicorius, Mingaile Drevinskaite, Marius Miglinas, Albertas Cekauskas, Morta Stadulyte, Rimante Bandzeviciute, Justinas Ceponkus, Valdas Sablinskas, Arunas Zelvys

**Affiliations:** a Faculty of Medicine, Institute of Clinical Medicine, Vilnius University, Vilnius, Lithuania; b Faculty of Physics, Institute of Chemical Physics, Vilnius University, Vilnius, Lithuania

**Keywords:** Urolithiasis, Stone composition, Spectroscopy, Stone analysis, Stone dust, Calcium oxalate stone, Uric acid stone

## Abstract

**Background:**

The introduction of the holmium laser for lithotripsy and minimally invasive techniques in endoscopy increased the popularity of stone dusting techniques. Retrieving stone pieces for an analysis increases the economic burden of surgery and operative time. Novel methods are needed for the analysis of convenient urolithiasis composition.

**Objective:**

This study aims to assess the efficacy of the stone dust Fourier transform infrared spectroscopy coupled with attenuated total reflection (FTIR ATR) method for accurate stone composition determination from the dust specimens compared with simultaneously retrieved standard stone fragments.

**Design, setting, and participants:**

From July 2021 to March 2022, a total of 75 patients who received endoscopic treatment for urolithiasis were included in this study.

**Outcome measurements and statistical analysis:**

The accuracy of the FTIR ATR method was assessed via estimates of sensitivity, specificity, negative predictive value (NPV), and positive predictive value (PPV). The results were compared between samples of stone dust and the final stone composition.

**Results and limitations:**

Total or partial biochemical composition agreement was observed in 92.7% of cases and total agreement in 82.4% of cases when stone dust was compared with stone fragments. The highest accuracy rates were obtained for uric acid stones: sensitivity 100%, specificity 98.3%, PPV 90.9%, and NPV 100%. Identification of other types of stones was also of high accuracy, reaching up to 83.3% sensitivity and 100% specificity.

**Conclusions:**

The application of FTIR ATR spectroscopy for a stone dust analysis allows obtaining easy and cost-effective final composition of urolithiasis without a stone fragment analysis. This technique was shown to be feasible, and there is substantial potential for clinical practice.

**Patient summary:**

This study investigates a novel method that determines accurate stone composition without acquiring the pieces of stone during surgery. The results have shown that stone dust Fourier transform infrared spectroscopy coupled with attenuated total reflection provides accurate stone composition.

## Take Home Message

Application of Fourier transform infrared spectroscopy coupled with attenuated total reflection for a stone dust analysis allows easy and cost-effective determination of the final composition of urolithiasis without a stone fragment analysis. This is a feasible technique, with suitable potential for future clinical practice.

## 1 Introduction

Urolithiasis is a common disease that affects 5–13% of the population worldwide [1]. It was observed that possibly due to dietary and lifestyle changes, the incidence of kidney stone disease has been increasing over the years, mainly in industrialized countries [2]. It has been reported that the increased prevalence of nephrolithiasis has caused higher surgical intervention rates for kidney stone management [3], [4]. The introduction of holmium laser for lithotripsy and minimally invasive techniques such as flexible ureteroscopy or mini percutaneous lithotripsy has become a backbone surgical treatment for kidney stone clearance.

The guidelines of the European Association of Urology recommend identification of the biochemical composition of kidney stones to understand the etiology and manage a recurrent stone disease [5]. However, in some clinical cases, it is impossible to get a stone fragment for a biochemical analysis; consequently, the stone dusting technique has become more popular in recent years [6], [7]. Urologists can use the stone dusting technique to break the stones into fine particles and expect the dust to pass spontaneously through the urinary tract. It is believed that this procedure is less traumatic and time consuming due to fewer passes with a ureteroscope [7].

Chemical spot tests for the biochemical composition of kidney stones are relatively inaccurate due to the false-positive and false-negative results, and cannot distinguish between the crystalline phases. The rapidly increasing number of reports are observed on the applications of the Fourier transform infrared (FTIR) spectroscopy coupled with attenuated total reflection (ATR) technique for the analysis of biofluids (saliva, blood, urine, etc.), tissues, and cells [8]. FTIR spectroscopy is well known for its ease of use, compact laboratory equipment, and low running costs [8].

The purpose of this study is to show the efficacy of a stone dust analysis for accurate determination of stone composition from the dust specimens compared against simultaneously retrieved standard stone fragments. The results contribute to the potential of FTIR ATR spectroscopy for the analysis of urine sediments.

## 2 Patients and methods

This prospective study was reviewed and accepted by the local ethical committee (no. 2019/3-1108-606). From July 2021 to March 2022, a total of 75 patients were included in the study. All patients underwent a computed tomography scan preoperatively and then endourological treatment: ureteroscopy, flexible ureteroscopy, or mini percutaneous lithotripsy. Surgeries were performed by eight different urologists. Holmium laser was the only technique used for stone dusting. Three types of samples were taken from every patient. The first sample of freshly voided urine (up to 50 ml) was collected before the operation (labeled as sample A in this study). The second sample of urine (labeled as sample B in this study) was taken during an endourological operation when stone dusting was performed. Urine (10–20 ml) with sediments was aspirated with a single-use syringe through an evacuation channel. The third and final sample was taken at the end of the operation, after several pieces of stones had been acquired for the final analysis (labeled as sample C in this study). In all cases, the irrigation fluid was normal saline. Patients were included in the study only if all samples were obtained.

FTIR spectrometer Alpha (Bruker Optik GmbH, Ettlingen, Germany) with a single-reflection diamond ATR module attached was used for the spectra collection. A glow-bar light source and a deuterated triglycine sulfate (DTGS) detector operating at room temperature were used in a standard spectrometer configuration. All the spectra were measured at 4 cm^–1^ resolution, in the spectral range between 400 and 4000 cm^1^. Each measurement consisted of 64 scans. Three-term Blackman-Harris apodization function, power spectrum phase correction mode, and zero filling factor of 2 were applied for Fourier transformation. After measurements, ATR correction, atmospheric compensation, and baseline correction for spectra were performed. The sample is placed and pressed on a diamond ATR crystal, and then an infrared (IR) beam is passed through a crystal at a total internal reflection angle. The evanescent wave of the IR beam is penetrating through the sample crystal interface. Typically, the penetration depth into the sample is several microns. This is the right thickness to obtain a proper IR absorption spectrum.

**Fig. 1:**
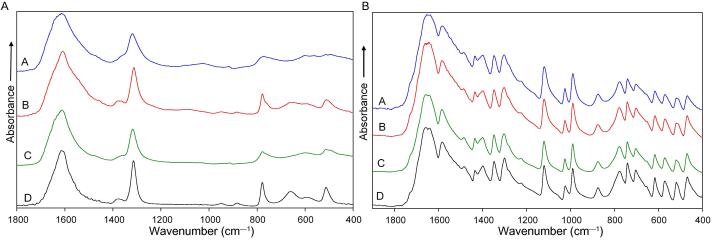
Patient sample IR absorption spectra of deposits of the urine taken before the surgery (line A), deposits of the urine taken during the surgery (line B), removed stone (line C), and reference spectrum of pure compound (line D). (A) all three samples contain calcium oxalate monohydrate. (B) All three samples contain uric acid. IR = infrared.

To prepare urinary deposits, urine samples A and B were centrifuged for 30 min at a speed of 12 000 rpm. Then the supernatant was removed, and the remaining deposits were placed on the ATR crystal and air dried for 5–10 min. Finally, ATR IR spectra of obtained nonorganic deposits were measured. Two to five measurements of different parts of each sample were performed. Sample measurements take about 3 min. If precipitated urinary deposits were covered in blood or protein, it was washed with distilled water and centrifuged for the second time for 10–20 min at 12 000 rpm speed before the measurements. Sample C (removed stone) was visually examined at first, and in cases where it contained areas of different colors or structure, all the distinguished parts were split from the stone and spectra of each split part were measured. All measured FTIR spectra of the samples were then compared against a library of spectra of pure possible chemical compounds. Typically, the time required for sample identification is within 1 h from the acquisition of the sample. The presence of a chemical compound in the examined sample was concluded from the visual comparison of the sample under investigation and the spectrum of the pure known chemical compound. If the spectral bands of the pure chemical compound were observed in the sample under investigation, the presence of that chemical compound is confirmed. Pure stones were defined as those having a single chemical compound in the whole stone, as shown in Figure 1.

**Fig. 2:**
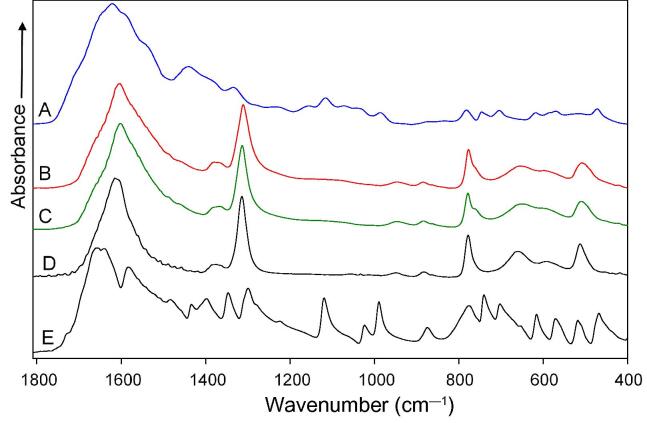
Sample before surgery consists of mixture of uric acid, proteins, and calcium oxalate, while sample during surgery and removed stone consist only of calcium oxalate; thus, dust and stone samples mach. Patient sample IR absorption spectra of deposits of the urine taken before the surgery (line A), deposits of the urine taken during the surgery (line B), removed stone (line C),reference spectrum of calcium oxalate (line D), and reference spectrum of uric acid (line E). IR = infrared.

A sample was defined as a mixed type if two different chemical compounds were detected in the stone or sediments. If the stone fragment and sediments from dust coincided, these were considered to be either both from the same compound or both mixed but composed of the same components. Results of the mixed sample type are illustrated in Figure 2.

Statistical analysis and graph plotting were performed with IBM SPSS Statistics 24.0 (IBM Corp, Armonk, NY, USA).

## 3 Results

Seventy-five patients participated in this study. However, only 68 (90.7%) of them were eligible for the final analysis. Forty-five (66.2%) patients were male and 23 (33.8%) female. The mean age was 60.48 ± 13.75 yr and the body mass index was 28.31 ± 4.54.

Forty-five (66.2%) patients underwent flexible or semirigid ureteroscopy operation and 23 (33.8%) mini percutaneous lithotripsy. The majority of the patients (73.5%) had a ureteral stent before the operation. The clinical characteristics of the patients are shown in Table 1.

**Table 1:** Clinical characteristics of the patients

Characteristic	Mean ± SD/*n* (%)
Age (yr)	60.48 ± 13.75
BMI (kg/m^2^)	28.31 ± 4.54
Type of operation	
URS or FURS	45 (66.2)
PCNL	23 (33.8)
Stone parameters on NCCT	
Maximum diameter of stone (mm)	15.56 ± 10.53
Mean Hounsfield unit score (HU)	857.03 ± 314.65
Highest Hounsfield unit score (HU)	1124.79 ± 411.42
Number of stones	
Single	31 (45.6)
Multiple	37 (54.4)
Extent of disease	
Unilateral	45 (66.2)
Bilateral	23 (33.8)

BMI = body mass index; FURS = flexible ureteroscopy; NCCT = noncontrast computed tomography; PCNL = percutaneous lithotripsy; SD = standard deviation; URS = ureteroscopy.

In 68 (90.7%) cases, urinary sediments were detected in the samples taken before the operation (samples A) or during the operation (samples B), or both of them, while the remaining specimens were insufficient for an FTIR ATR spectroscopy analysis. Ten of 75 stone dust (B group) samples and 19 of 75 preoperatively taken urine (A group) samples did not have enough sediments for an analysis.

**Fig. 3:**
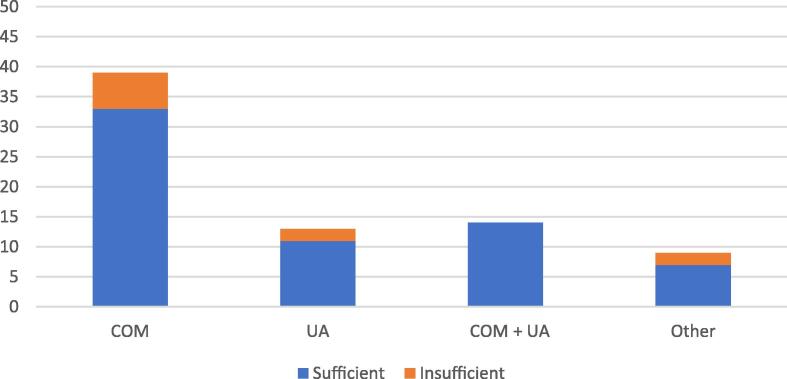
Types of final stone composition when lasered stone dust was sufficient (*n* = 65) and insufficient (*n* = 10) for an analysis by FTIR ATR spectroscopy. COM = calcium oxalate monohydrate; COM + UA = calcium oxalate monohydrate mixed with uric acid; FTIR ATR = Fourier transform infrared spectroscopy coupled with attenuated total reflection; Other = struvite, carbonate apatite, and calcium phosphate; UA = uric acid.

Different types of stones produce different quantities of dust. From ten stone dust samples that could not be analyzed by FTIR ATR spectroscopy, six (60%) were retrieved when stones of calcium oxalate monohydrate (COM) type were dusted. Only two (20%) samples were from uric acid stones. This indicates that sample quality could be affected by the type of stone dusted during operation (Fig. 3).

Further calculations revealed that cases with sufficient dust samples had different characteristics of stones on noncontrast computed tomography (NCCT) than insufficient ones. The maximum diameter of a stone was higher in such cases (7.85 + 2.27 vs 16.04 + 10.66 mm, *p* < 0.05). However, a further regression analysis displayed that NCCT parameters (maximum diameter, Hounsfield unit density, and highest score) did not have a clinically significant impact on dust sample quality.

The most accurate prediction of final stone composition was observed when group B samples (samples taken during stone dusting) were compared with group C samples (pieces of the stone). In 63 (92.7%) of 68 cases, IR spectroscopy results were in total or partial agreement with the final stone composition. The total agreement was reached in 56 (82.4%) cases. Group A samples (urine taken before operation) were in disagreement with final stone composition in 33 (48.5%) cases, partial agreement in 11 (16.2%) cases, and total agreement in 24 (35.3%) cases. No further analysis was performed on group A samples due to the imprecision of the results. It was decided that the method that has agreement only in around 50% of cases should not be used in a clinical practice. Table 2 summarizes observations from the further comparison between sample groups.

**Table 2:** Agreement between the results of urinary sediment compositions of different sample groups

Comparison between groups	Agreement, *n* (%)	Partial agreement, *n* (%)	Disagreement, *n* (%)
A with B	24 (35.3)	5 (7.4)	39 (57.4)
B with C	56 (82.4)	7 (10.3)	5 (7.4)
A with C	24 (35.3)	11 (16.2)	33 (48.5)

A = urinary samples taken before operation; B = urinary samples taken during stone dusting; C = pieces of stone taken during or after the operation.

**Fig. 4:**
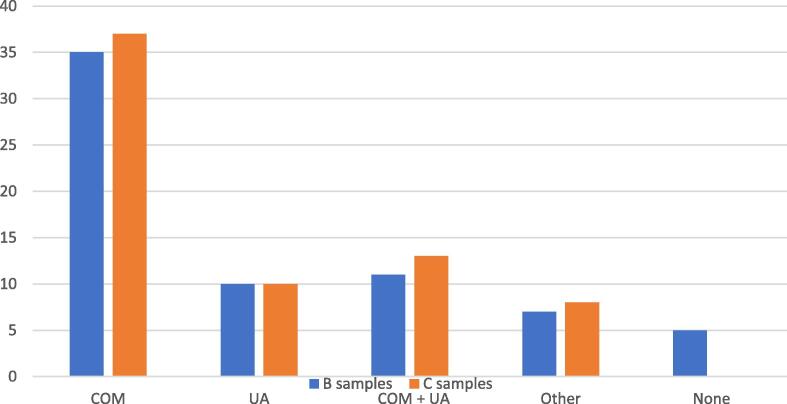
Types of urinary sediments in stone dust (samples B) and final stone composition (samples C). COM = calcium oxalate monohydrate; COM + UA = calcium oxalate monohydrate mixed with uric acid; Other = struvite, carbonate apatite, and calcium phosphate; UA = uric acid.

Compounds found in the stone composition (samples C) by an FTIR ATR analysis were very similar to those in the composition of stone dust (samples B). The most common sediment was pure COM, accounting for 37 (54.4%) samples C. Pure uric acid was found in ten (14.7%) samples, mixed type of sediments (COM with uric acid) were found in 13 (19.1%) samples, and other sediments such as struvite, calcium phosphate, and carbonate apatite were found in eight (11.8%) samples (Fig. 4).

In the end, the accuracy of the FTIR ATR method was evaluated by comparing the composition of stone dust and removed stone. The sensitivity, specificity, positive predictive value, and negative predictive value were calculated. The highest accuracy rates were obtained for uric acid stones (sensitivity 100% and specificity 98.3%). Identification of other types of stones was also of high accuracy, reaching up to 83.3% sensitivity and 100% specificity. The final results are presented in Table 3.

**Table 3:** Sensitivity (Sens.), specificity (Spec.), positive predictive value (PPV), and negative predictive value (NPV) of FTIR of stone dust, when FTIR of the paired stone fragment is the standard for each stone type assessed

Type of dust	No. of cases	Sens. (%)	Spec. (%)	PPV (%)	NPV (%)
COM	37	83.3	87.5	88.2	82.4
UA	10	100	98.3	90.9	100
COM + UA	13	69.2	96.4	81.8	93
Other	8	75	100	100	96.8
Total or median	68	81.8	95.6	90.2	93.1

COM = calcium oxalate monohydrate; COM + UA = calcium oxalate monohydrate mixed with uric acid; FTIR = Fourier transform infrared; Other = struvite, carbonate apatite, and calcium phosphate; UA = uric acid.

## 4 Discussion

This prospective study demonstrates the successful results of the ability of FTIR ATR spectroscopy to establish the biochemical composition of stone from the dust compared with the standard stone fragments.

Several methods, such as chemical analysis, chromatography, polarized light microscopy, scanning electron microscopy, Raman scattering spectroscopy, or FTIR spectroscopy, can be used for the determination of a stone’s chemical composition. The application of a chemical analysis requires performing various chemical reactions to determine the compounds of the stone. Although this technique is not expensive and does not require special equipment, its use is limited by the fact that some of the components of the stone cannot be detected due to their insolubility, and it is not possible to distinguish between materials of similar composition [9]. Comparatively, ion chromatography is a sensitive and accurate method, and requires only a small amount of sample for analysis, but the analysis is expensive and complex [10]. Polarized light microscopy is also a sensitive method requiring a small amount of sample, but it is unsuitable for the investigation of amorphous or fine-grained materials. While scanning electron microscopy reveals complex information about the sample, the equipment is large and highly expensive. Raman scattering spectroscopy provides relevant information about the sample composition but is less sensitive and more expensive than ATR FTIR spectroscopy [9]. In this study, ATR FTIR spectroscopy was applied. This technique makes sample preparation simple, and the spectrum, which contains information about the composition of the sample, can be measured quickly from a small amount of material. Whereas spectral libraries can be used for automated spectral analysis and sample composition determination, data analysis does not require specific knowledge and skills in spectroscopy. Regarding all the advantages mentioned, ATR FTIR spectroscopy can easily be used for the identification of urinary stone composition [11].

The authors of this study believe that some of the samples did not contain urine sediment due to improper sampling techniques or various factors that may have influenced the composition of the urine sample and thus affected the final result. The success of good sample acquisition may be improved by increasing the quality of the dust specimen. A good sample requires at least 0.1 mg of dust material. If there is visible dust at the bottom of the container, this is often sufficient. Procedures where larger stones are dusted create more visible dust clouds, making it easier to get a suitable dust sample. It is particularly important to close the irrigation channel before specimen aspiration with a syringe. If irrigation is still ongoing during the sample aspiration, then there is a high chance of retrieving fluid from the irrigation system but not from the urinary system. Another particularly important factor is blood: specimen heavily contaminated with blood often results in incorrect estimations. A high amount of protein results in very strong IR spectral bands, prohibiting the detection of weak bands from small amounts of sediments.

In our study, we observed a total biochemical composition agreement in 82.4% of cases when the composition of stone dust and stone fragments was compared using FTIR spectroscopy. To our knowledge, the first study by Ray et al [12], in which authors compared the biochemical composition of stone dust and stone fragments using FTIR spectroscopy, found 74% of sufficient dust specimens to match fragmented stone composition. The majority of extracted stones (dust or stone fragments) were composed of pure COM (54.4%), and 10.7% of stones were of uric acid. However, the results were noticeably better for uric acid stones. We found out that the sensitivity reached 100%, specificity 98%, positive predictive value 90.9%, and negative predictive value 100% in the uric acid stones’ group. In the mentioned study [12], authors obtained similar results to ours: the sensitivity in the uric acid stones’ group was 90.9%, specificity and positive predictive values were 100%, and negative predictive value was 98.9%. The authors discussed that better results could have been attributed to the biochemical qualities of different uric acid stones compared with other stones. Owing to the soft and pure homogeneous nature of uric acid stones, a larger dust cloud is seen and collected during the laser treatment procedure [13], [14]. In the COM group, results were slightly different with sensitivity of 83.3%, specificity of 87.5%, and positive and negative predictive values reaching >80%. Interestingly, the sensitivity and positive predictive value (69.2% and 81.8, respectively) were worse, and specificity and negative predictive value were better (96.4% and 93%, respectively) in the group of mixed stones of COM and uric acid compared with pure COM stones. In the same group of biochemically mixed stones, Ray et al [12] reported 100% sensitivity, specificity, positive predictive value, and negative predictive value. On the contrary, it should be noted that they examined only one case of mixed calcium oxalate and uric acid stone, while we describe 13 cases in our study.

The kidney stone dusting technique is thought to be less time consuming and, consequently, less costly because multiple passes with a ureteroscope and basket retrieval devices are unnecessary [7]. In our study, we have not compared the financial burden of different techniques, due to the fact that the usage of a basket retrieval device to extract stone fragments and dusting the stone residues were applied during the same surgery. However, Humphreys et al [15], in their multi-institutional, randomized study, in which they evaluated basketing and dusting techniques, observed that dusting was associated with a 44% reduction in operative time, which should translate to reduced operative cost. Moreover, another study estimated that for ureteroscopic lithotripsy, 74% of procedure resources depend on operative time, emphasizing the potential financial impact of the technique [16].

Our study has several limitations that should be stated. First, in this prospective study, eight different surgeons performed kidney stone surgical procedures (kidney stone fragmentation, basketing, and then dusting). We believe that there could be a possibility of inherent biases. Second, a relatively small number of cases were analyzed. Last, more types of stones should be analyzed. In our study, we determined only COM, uric acid, mixed, struvite, calcium phosphate, and carbonate apatite types of stones. Future studies should include studying the effect of stone layers by a chemical analysis, and the effect of different laser types or settings on the quality of the dust specimen.

## 5 Conclusions

Our study contributes to the existing information that the FTIR ATR spectroscopy method could be used as a primary diagnostic tool for final stone composition when stone dusting is performed without using a basket, which increases the economic burden of the operation. In this pilot study, we have assessed the feasibility of collecting stone dust and the accuracy of measurement of stone composition from dust specimens compared with simultaneously retrieved standard stone fragments. We observed total or partial biochemical composition agreement in 92.7% of cases, and total agreement in 82.4% of cases. In our opinion, application of FTIR ATR spectroscopy for a stone dust analysis allows easy and cost-effective determination of the final composition of urolithiasis without a stone to analyze. This innovative method could be the future of a urolithiasis analysis, especially when stone dusting is getting more and more popular among endoscopic lithotripsy techniques. Further advances in the FTIR ATR methodology could shorten sample preparation time, and such an analysis could provide information about urolithiasis components within surgery or during patients’ stay at the hospital for surgery. This is a feasible technique, with suitable potential for future clinical practice.

  ***Author contributions*:** Marius Snicorius had full access to all the data in the study and takes responsibility for the integrity of the data and the accuracy of the data analysis.

  *Study concept and design*: Snicorius, Drevinskaite, Zelvys, Sablinskas.

*Acquisition of data*: Snicorius, Stadulyte, Bandzeviciute, Ceponkus.

*Analysis and interpretation of data*: Snicorius, Stadulyte, Bandzeviciute, Ceponkus.

*Drafting of the manuscript*: Snicorius, Stadulyte, Drevinskaite.

*Critical revision of the manuscript for important intellectual content*: Miglinas, Cekauskas, Sablinskas, Zelvys.

*Statistical analysis*: Snicorius.

*Obtaining funding*: None.

*Administrative, technical, or material support*: Zelvys, Cekauskas, Miglinas, Sablinskas.

*Supervision*: Zelvys, Sablinskas.

*Other*: None.

  ***Financial disclosures:*** Marius Snicorius certifies that all conflicts of interest, including specific financial interests and relationships and affiliations relevant to the subject matter or materials discussed in the manuscript (eg, employment/affiliation, grants or funding, consultancies, honoraria, stock ownership or options, expert testimony, royalties, or patents filed, received, or pending), are the following: None.

  ***Funding/Support and role of the sponsor*:** None.

  ***Acknowledgments*:** The authors would like to thank all the patients who participated in this study for their important contributions.
